# An Early Trial‐Based Cost‐Effectiveness Analysis of Extracorporeal Shockwave Therapy for Diabetes Related Foot Ulcer Healing

**DOI:** 10.1111/iwj.70925

**Published:** 2026-05-24

**Authors:** L. Hitchman, M. Siciliano, R. Lathan, B. Ravindhran, M. Sidapra, J. Long, G. Smith, M. Twiddy, D. Russell, I. C. Chetter, C. Iglesias Urrutia

**Affiliations:** ^1^ Faculty of Clinical Sciences Hull York Medical School Hull UK; ^2^ Department of Health Sciences University of York York UK; ^3^ Hull University Teaching Hospitals NHS Trust Hull UK; ^4^ Institute of Clinical and Applied Health Research, Hull York Medical School Hull UK; ^5^ Leeds Vascular Institute, Leeds Teaching Hospitals NHS Trust Leeds UK; ^6^ Leeds Institute of Clinical Trials Research, University of Leeds Leeds UK

**Keywords:** cost‐effectiveness, diabetic foot ulcers, economic evaluation, extracorporeal shockwave therapy, wound healing

## Abstract

Diabetes related foot ulcers (DFUs) cause significant morbidity and mortality. Extracorporeal shockwave therapy (ESWT) is a promising adjunct to promote healing. This study aimed to explore the potential cost‐effectiveness of ESWT in DFU healing. Early model‐based cost‐effectiveness analysis was undertaken. A de novo four‐state, 1‐day cycle, Markov model was developed. The perspective for the analysis was the UK's National Health Service (NHS) and Personal Social Services over a 1‐year time horizon. Input parameters were estimated from the literature. Low dose ESWT, high dose ESWT and standard care alone were evaluated by running a simulation of 1000 patients with one DFU. Deterministic, probabilistic and value of information analysis were undertaken. Deterministic analysis indicated high dose ESWT has the potential to be a dominant strategy (i.e., no incremental analysis was necessary), exhibiting higher QALYs and lower costs compared to low dose ESWT and standard care. The mean difference in QALYs and costs between high dose ESWT and standard care were 0.022 QALYs and a £922 cost reduction. Preliminary probabilistic analysis, using a threshold of £20 000 per QALY, estimated a 60.2% probability of high dose ESWT being cost‐effective compared to standard care. The value of eliminating uncertainty in the model (EVPI) was estimated at £24.56 per patient, with high dose efficacy (partial EVPI per patient £7.87) and health‐related quality of life (HRQoL) scores (partial EVPI per patient £3.56) identified as specific areas for future research. This study suggests that ESWT could be a dominant adjunct to aid DFU healing. A definitive study is warranted to estimate ESWT's effectiveness and related HRQoL scores.

## Key Messages

1. High dose ESWT has the potential to dominate standard of care alone and low dose ESWT to aid DFU healing.
2. Preliminary results indicate the chance of high dose ESWT being cost effective compared to standard of care may be 60%.
3. The preliminary maximum value of conducting more research to estimate ESWT's clinical and cost effectiveness for the population of England with one new DFU is estimated to be between £1 289 906 and £2 579 813 (population EVPI).
4. A definitive study is warranted to reduce current uncertainty with the effectiveness of ESWT and related HRQoL scores.

## 1 Background

The worldwide prevalence of diabetes related foot ulcers (DFUs) in adults with diabetes is estimated between 0.9% and 53.9% [1]. Non healing DFUs can lead to significant complications resulting in limb loss, increased mortality and a reduced health‐related quality‐of‐life [2, 3]. There is a growing epidemic of diabetes globally, which is causing a significant strain on healthcare resources [1]. Currently the National Health Service (NHS) in England spends an estimated £1 billion annually managing complications of DFUs and between $11–17 billion is spent in the USA [4, 5].

The mainstay of treatment of DFUs is regular debridement, offloading footwear, glycaemic control, dressing changes, control of infection and restoration of lower limb perfusion [6, 7, 8, 9, 10]. Despite this, 10%–18% of DFUs result in a major lower limb amputation each year and the estimated mortality rate is 14.4% [11, 12, 13]. There is a plethora of adjuncts designed to reduce DFUs healing time and number of complications. However, many do not progress into routine clinical practice due to lack of high‐quality evidence supporting their clinical and cost‐effectiveness [6].

Extracorporeal shockwave therapy (ESWT) is proposed to improve wound healing by using low frequency soundwaves to stimulate healing pathways and neovascularisation [14, 15, 16, 17]. The intervention is acceptable to patients and deliverable at the bedside or in an outpatient clinic [18]. ESWT involves placing a gel paddle onto a prepared wound bed that is covered with a sterile film and discharging shockwaves into the wound. Emerging evidence supports the effectiveness of ESWT to accelerate DFU healing time, but the optimal dose of shockwaves needed is unclear [19, 20]. A pilot randomised controlled trial (RCT) in England was undertaken investigating two different doses of ESWT in patients with DFUs present for 4 weeks or longer. The SOLEFUL trial recruited 74 patients who, in addition to standard DFU care, were randomised to either low dose ESWT (100 shocks/cm^2^), high dose ESWT (500 shocks/cm^2^) or sham ESWT (0 shocks/cm^2^). ESWT was delivered three times over a 7‐day period and patients were followed up for 6 months [21].

The purpose of this study was to conduct an early trial‐based cost‐effectiveness analysis, populated with data from the SOLEFUL pilot trial, to explore whether ESWT had the potential to be a cost‐effective intervention for use in the NHS for patients with non‐healing DFUs.

## 2 Methods

A decision analytic model was used to simulate a cohort of patients through different health states relevant to DFUs. The analysis was undertaken following the principles outlined by the Professional Society for Health Economics and Outcomes Research (ISPOR) [22, 23, 24] and is reported with reference to the Consolidated Health Economic Evaluation Reporting Standards (CHEERS) checklist and the CHEERS‐value of information (VOI) checklist [25, 26].

The aim of the model was to explore the cost‐effectiveness of ESWT in reducing DFU healing time. The objective was to quantify the difference in costs and Quality Adjusted Life Years (QALYs) of low dose and high dose ESWT in addition to standard ulcer care compared to standard ulcer care alone.

### 2.1 Model Structure

The decision problem focused on the following areas:

#### 2.1.1 Cohort Population

A simulated group of 1000 patients with neuropathic and neuro‐ischaemic DFUs treated within the NHS outpatient secondary care setting. The cohort population was based on the SOLEFUL trial participants [21].

#### 2.1.2 Technology

Two doses of ESWT were evaluated: low dose (100 shock/cm^2^) and high dose (500 shocks/cm^2^). Linear focused shockwaves were given at an energy influx of 0.16 mJ/mm^2^ and a frequency of 5 Hz. The trial which this evaluation is based upon used the Richard Wolfe PiezoWave^2^ device [21].

Analysis was based on ESWT being delivered three times in 1 week in the secondary healthcare setting by a band 6 podiatrist. ESWT is in addition to standard DFU care.

#### 2.1.3 Comparator

Standard DFU care, consisting of glycaemic control, debridement of the ulcer, offloading, infection control, regular dressing changes and, when necessary, revascularisation, is delivered by the members of the DFU multidisciplinary team.

#### 2.1.4 The Model

**FIGURE 1:**
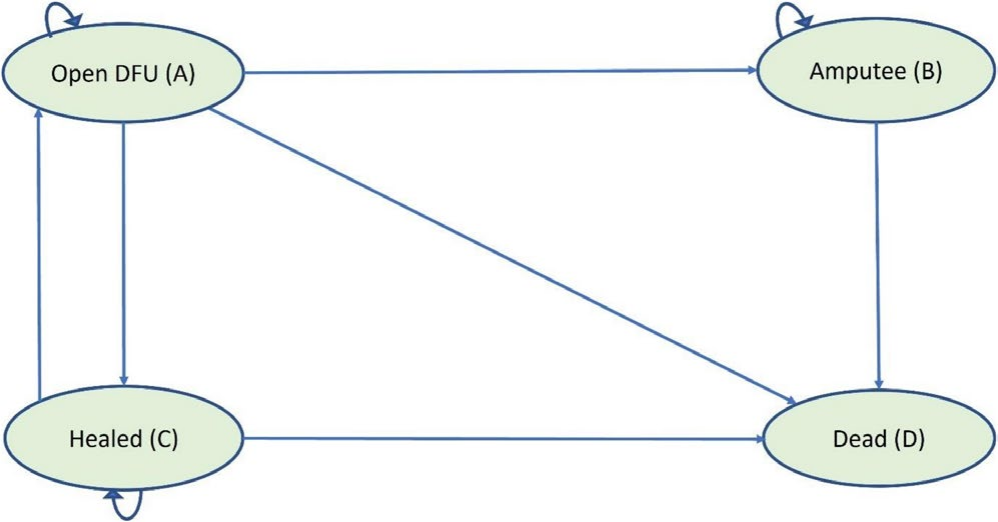
Model structure.

A Markov state transition model was used (Figure 1). This allowed for the natural history of DFUs to be represented in a manageable number of states with relevant characteristics to represent the decision problem. The model structure was based on existing decision models of diabetic foot disease [27, 28, 29, 30, 31, 32, 33, 34, 35, 36, 37, 38, 39, 40, 41, 42, 43, 44, 45, 46, 47, 48, 49, 50]. A subgroup analysis was conducted to explore the impact of ulcer characteristics known to impact healing, including variations in ulcer duration, size and infection status [22, 51]. The model included four mutually exclusive [24] health states corresponding with the underlying observed disease process:

○ Open DFU (A): a DFU. This includes patients living with diabetes who have an open wound on the foot present for more than 4 weeks
○ Amputee (B): Following an ipsilateral major lower limb amputation (MLLA). There is no further risk of ulceration as the leg has been removed.
○ Healed (C): a healed DFU, defined as complete healing for at least 2 weeks
○ Dead (D): the person is deceased

The model starts with a cohort of 1000 people with an unhealed DFU. Transition probabilities were applied after a 1‐day cycle length.

#### 2.1.5 Cycle Length

The model is based on the SOLEFUL pilot trial data, where time of healing, MLLA and death are known to the nearest day [21]. Thus, we used a 1‐day cycle length to take full advantage of our data. Given the short cycle length, no half‐cycle correction was needed.

#### 2.1.6 Time Horizon

A 1‐year time horizon was used. It is highly unlikely that ESWT will influence events after 1 year. Patients are modelled per ulceration episode, rather than their lifetime risk of developing recurrent DFUs. Discounting was not applied as the time horizon was 1 year.

#### 2.1.7 Perspective

The perspective of the economic evaluation was from the UK's NHS and Personal Social Services.

#### 2.1.8 Model Inputs

The model inputs were based on data collected during the SOLEFUL pilot trial [21] and current literature. Transition probabilities of healing, recurrence, amputee and death were converted into an instantaneous rate and then into a daily transition probability [52]. The impact of ESWT on transition probabilities was based on the SOLEFUL pilot trial, rather than results from a meta‐analysis. This is because a recent systematic review has recommended data from existing trials should not be combined to compare different doses of ESWT due to high‐risk bias and low quality of current evidence [19]. No participants died during the 6‐month follow‐up period; data on mortality was extracted from the literature. Death in a healed health state was based on an 11 000‐patient cohort study conducted in Salford, Manchester, UK [53]. Death rates with an open DFU and following a MLLA were based on a systematic review [12]. The daily transition probabilities are shown in Table 1.

**TABLE 1:** Transition probabilities.

From open DFU to	Daily transition probability
Healed DFU	Survival analysis
Amputee	0.00308674
Dead	0.00019921
Open DFU	1 − (survival analysis healing − 0.00308674 − 0.00019921)
From amputee to	
Dead	0.000456958
Amputee	1 − 0.000456958
From healed DFU	
DFU recurrence	0.00308674
Dead	6.0435E‐05
Healed DFU	1 − (0.00308674 − 6.0435E‐05)
From dead to	
Dead	1

Abbreviation: DFU, diabetes related foot ulcer.

#### 2.1.9 Utilities

The utility values were estimated from the SOLEFUL pilot trial population [21]. EQ‐5D‐5L was used to collect the health‐related quality‐of‐life scores and the UK value set was used to assign utility values [54]. The mean utility values for the three health states of open DFU, amputee and healed DFU were respectively 0.671, 0.316 and 0.757.

#### 2.1.10 Costs

Healthcare resource use was based on the SOLEFUL trial results [21] and costs were estimated from the NHS reference costs, the British National Formulary (BNF) and the Personal Social Services Research Unit (PSSRU) using 2022/2023 prices [55, 56, 57]. The included costs are reported in Table 2. The daily costs for each health state were estimated as £15.01 for state A (open DFU), £22.64 for state B (amputee) and £1.89 for state C (healed DFU).

**TABLE 2:** Healthcare resource cost sources.

Health resource use	Data source
Podiatry appointments	National schedule of NHS Costs—non‐consultant podiatry led non‐admitted face‐to‐face, follow up Number of appointments recorded in the CRF
Diabetic Foot MDT appointments	National Schedule of NHS Costs—consultant led vascular surgery multiprofessional non‐admitted face‐to‐face attendance, first and follow up Number of appointments recorded in the CRF
Hospitalisation	Based on national schedule of NHS costs Elective inpatients: − Diabetes with lower limb complications, with CC Score 5–8 − Amputation of single limb with CC Score 0–9 − Single, amputation stump or partial foot amputation procedure, for diabetes or arterial disease, with CC Score 5–7 − Peripheral vascular disorders with CC Score 0–1 − Percutaneous transluminal angioplasty of multiple blood vessels with CC Score 0–2 Non‐elective inpatients: − Diabetes with lower limb complications, with CC Score 5–8 − Diabetes with lower limb complications, with CC Score 0–4 − Single open procedure on blood vessel of lower limb with CC Score 0–3 − Amputation of single limb with CC Score 0–9 − Single, amputation stump or partial foot amputation procedure, for diabetes or arterial disease, with CC Score 5–7 − Single, amputation stump or partial foot amputation procedure, for diabetes or arterial disease, with CC Score 0–4 − Peripheral vascular disorders with CC Score 5–7 − Peripheral vascular disorders with CC Score 2–4 − Percutaneous transluminal angioplasty of multiple blood vessels with CC Score 3–5 − Percutaneous transluminal angioplasty of multiple blood vessels with CC Score 0–2 − Percutaneous transluminal angioplasty of single blood vessel with CC Score 0–2 − Surgical adult patients (unspecified speciality)—adult critical care, 1 organ supported Emergency care: − Emergency Medicine, Category 2 investigation with Category 3 treatment Number of events recorded in SOLEFUL pilot trial serious adverse event log
GP appointments	PPSRU 9.22 min consultation with a GP, excluding qualification costs and including direct staff care costs Number of appointments recorded in the CRF
District nursing visits	PSSRU 30 min visit from a band 6 nurse Number of appointments recorded in the CRF
Practice nurse visits	PSSRU 15 min consultation with a band 6 nurse Number of appointments recorded in the CRF
Dressings cost	BNF 2022 prices

Abbreviations: BNF, British national formulary; CRF, case report form; GP, general practitioner; MDT, multidisciplinary team; NHS, National health service; PSSRU, Personal Social Services Research Unit.

### 2.2 Analysis

Analyses were carried out using Microsoft Excel (WA, USA) and STATA (Version 18; TX, USA).

#### 2.2.1 Time to Healing

The survival data from the trial was modelled using the Weibull distribution. This distribution was chosen by examining the plots of different model distribution survival functions, comparing the smoothed hazard function with the fitted hazard function and comparing the Akaike Information Criterion (AIC) [58].

A Weibull regression analysis was used to explore the influence of the following covariates on healing: low dose ESWT, high dose ESWT, age of the DFU, surface area of the DFU and infection status. The Cholesky decomposition was then applied to the regression model from the covariance matrix.

#### 2.2.2 Base Case Analysis

A deterministic base case analysis was undertaken to estimate the cost and QALYs of standard care, low dose ESWT and high dose ESWT.

#### 2.2.3 Decision Rule

The decision rule was based on the difference in QALYs and costs between treatment strategies. Standard rules of cost‐effectiveness were used to interpret our preliminary findings on value for money. An alternative was considered dominant if it was associated with more QALYs but was less costly. QALY is a measure of health utility. It measures quality of life over time, where 1 year in perfect health equals 1 QALY [59].

#### 2.2.4 Probabilistic Sensitivity Analysis

Using the results from the Weibull regression and Cholesky decomposition, a probabilistic sensitivity analysis was undertaken. Input parameters were drawn from beta distributions for the transition probabilities, log‐normal distributions for the hazard ratios and gamma distributions for QALYs and costs. All distribution parameters can be found in Table 3 [52].

**TABLE 3:** Model parameters and distributions [52].

Parameter	Distribution
Transition probabilities	
Healing	Beta (survival analysis)
Amputee	Beta (0.0308674, 99.9691326)
Recurrence	Beta (0.0308674, 99.9691326)
Mortality with a DFU	Beta (0.019921, 99.980079)
Mortality with a healed DFU	Beta (0.0060453, 99.9939547)
Mortality with amputee	Beta (0.0456958, 99.9543042)
Healing rates	
Constant	Log normal (survival analysis)
Age of the DFU	Log normal (survival analysis)
Area of the DFU	Log normal (survival analysis)
Infection status of the DFU	Log normal (survival analysis)
Lambda	Log normal (survival analysis)
Gamma	Log normal (survival analysis)
Low dose ESWT	Log normal (survival analysis)
High dose ESWT	Log normal (survival analysis)
Costs	
Open DFU	Gamma (49.0, 0.30623907)
Healed DFU	Gamma (49.0, 0.00387172)
Amputee	Gamma (49.0, 0.46201166)
Utilities	
Open DFU	Beta (47.73048902, 15.3633487)
Amputee	Beta (0.647532157, 1.39838303)
Healed DFU	Beta (130.5409719, 64.1219746)

Abbreviation: DFU, diabetes related foot ulcer.

Results are presented at a ceiling ratio of £20 000 as this represents NICE's willingness‐to‐pay threshold [60].

#### 2.2.5 Subgroup Analysis

A subgroup analysis was performed by changing DFU size, duration and infection status. Results are presented as net monetary benefit (NMB) at a ceiling ratio of £20 000.

#### 2.2.6 Value of Perfect Information

A VOI analysis was undertaken to estimate preliminary expected individual and population value of perfect information (EVPI); and, per patient expected value of partial perfect information (EVPPI). The EVPI describes the maximum monetary value of reducing decision uncertainty. Consequently, the EVPI is often interpreted as the maximum cost that should be spent on further research [61]. A simulation approach was used to calculate the EVPI. In each iteration, the opportunity loss is equivalent to the difference between the NMB under perfect information and the NMB under current information. The opportunity loss from all iterations was then averaged to compute the per‐patient EVPI [52].

We computed the EVPI to preliminary estimate the value of eliminating uncertainty for all patients who could potentially benefit from ESWT. This target population was modelled for 1 year with an incidence of DFU between 50 000 and 100 000 per year [62, 63]. A range was used due to the unclear incidence of DFU in England. The population EVPI was computed and plotted for different willingness‐to‐pay thresholds to estimate the value for money of undertaking future research and a potential upper limit for additional research funding at different thresholds per QALY [52].

To explore which model parameters carried the most uncertainty and where further research should focus [52, 64], we computed the EVPPI for the following key variables: risk of healing with high dose ESWT, daily state‐specific costs and health state‐related utilities (healed DFU, non‐healing DFU and amputee). The EVPPI was calculated using the Claxton formulation [65]. We followed a nested simulation approach to compute the EVPPI, using 1000 inner loops and 250 outer loops to keep manageable execution times [52, 66].

### 2.3 Ethics

The SOLEFUL study, which encompasses a pilot RCT, qualitative study and economic evaluation, was approved by the NHS Health Research Authority (IRAS: 311664, REC reference: 22/WA/0089) and sponsored by the Hull University Teaching Hospitals NHS Trust. The study was undertaken in line with the Declaration of Helsinki [67].

## 3 Results

### 3.1 Deterministic Modelling

High dose ESWT and low dose ESWT were both associated with an improved quality of life and lower costs compared to standard care alone. High ESWT was the dominant treatment strategy (Table 4). Low dose ESWT was therefore excluded from further analysis. The mean per‐patient costs and QALYs for standard care were £4538.48 and 0.650, respectively, compared to £3616.81 and 0.672 QALYs for high‐dose ESWT. High dose ESWT was £921.67 cheaper than standard care. The difference in QALYs was 0.022.

**TABLE 4:** Preliminary results of the deterministic analysis.

	Cost	Mean QALYs per patient at 12 months	Cost difference (vs. standard care)	QALY difference (vs. standard care)
Standard care	£4538.48	0.650		
High dose ESWT	£3616.81	0.672	−£921.67	0.022
Low dose ESWT	£4097.10	0.660	−£441.37	0.011

Abbreviations: ESWT, extracorporeal shockwave therapy; QALY, quality adjusted life year.

### 3.2 Probabilistic Modelling

**FIGURE 2:**
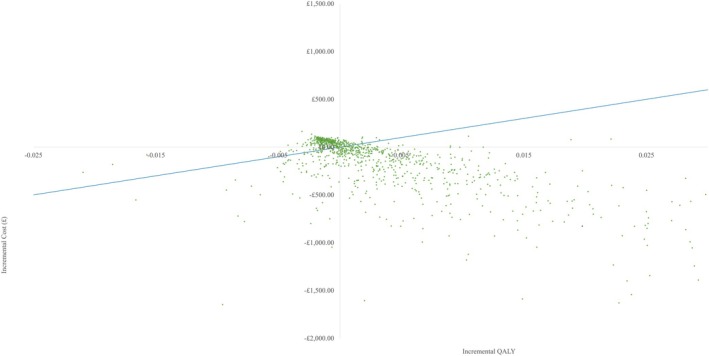
Incremental cost‐effectiveness plane for high dose ESWT compared to standard of care. The red dot in the red circle is the mean incremental cost and QALY. The blue line is the £20 000 willingness to pay threshold.

**FIGURE 3:**
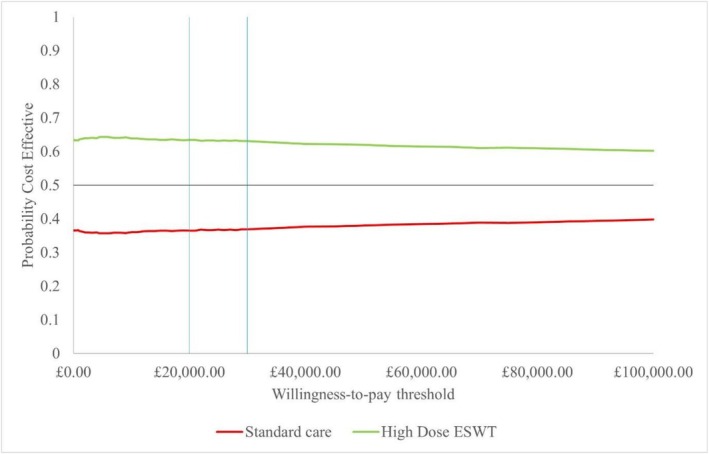
Cost‐effectiveness acceptability curve. Blue vertical lines = willingness to pay threshold at £20 000 and £30 000; black horizon line = 50% probability of being cost‐effective.

In the probabilistic sensitivity analysis, which ran 1000 simulations at the £20 000 per QALY threshold, high dose ESWT was cost‐effective compared to standard of care in 60.2% of the simulations (Figures 2 and 3).

The potential cost‐effectiveness of ESWT was further explored in 12 subgroups with different DFU characteristics, at a willingness‐to‐pay threshold of £20 000 per QALY (Table 5). The greatest NMB was associated with treating DFUs ≤ 1cm^2^, present for 4 weeks, regardless of infection status. The high dose ESWT treatment strategy had the highest NMB for all subgroups.

**TABLE 5:** Subgroup analysis.

Scenario	High dose ESWT, NMB	Low dose ESWT, NMB
Infected DFU DFU area: 1 cm^2^ DFU duration: 30 days	£806.00	£348.64
Infected DFU DFU area: 5 cm^2^ DFU duration: 90 days	£601.50	£241.77
Infected DFU DFU area: 10 cm^2^ DFU duration: 180 days	£390.30	£130.28
Non infected DFU DFU area: 1 cm^2^ DFU duration: 30 days	£615.28	£230.07
Non infected DFU DFU area: 5 cm^2^ DFU duration: 90 days	£448.41	£158.14
Non infected DFU DFU area: 10 cm^2^ DFU duration: 180 days	£236.03	£45.36

Abbreviations: DFU, diabetes related foot ulcer; ESWT, extracorporeal shockwave therapy; NMB, net monetary benefit.

### 3.3 VOI

**FIGURE 4:**
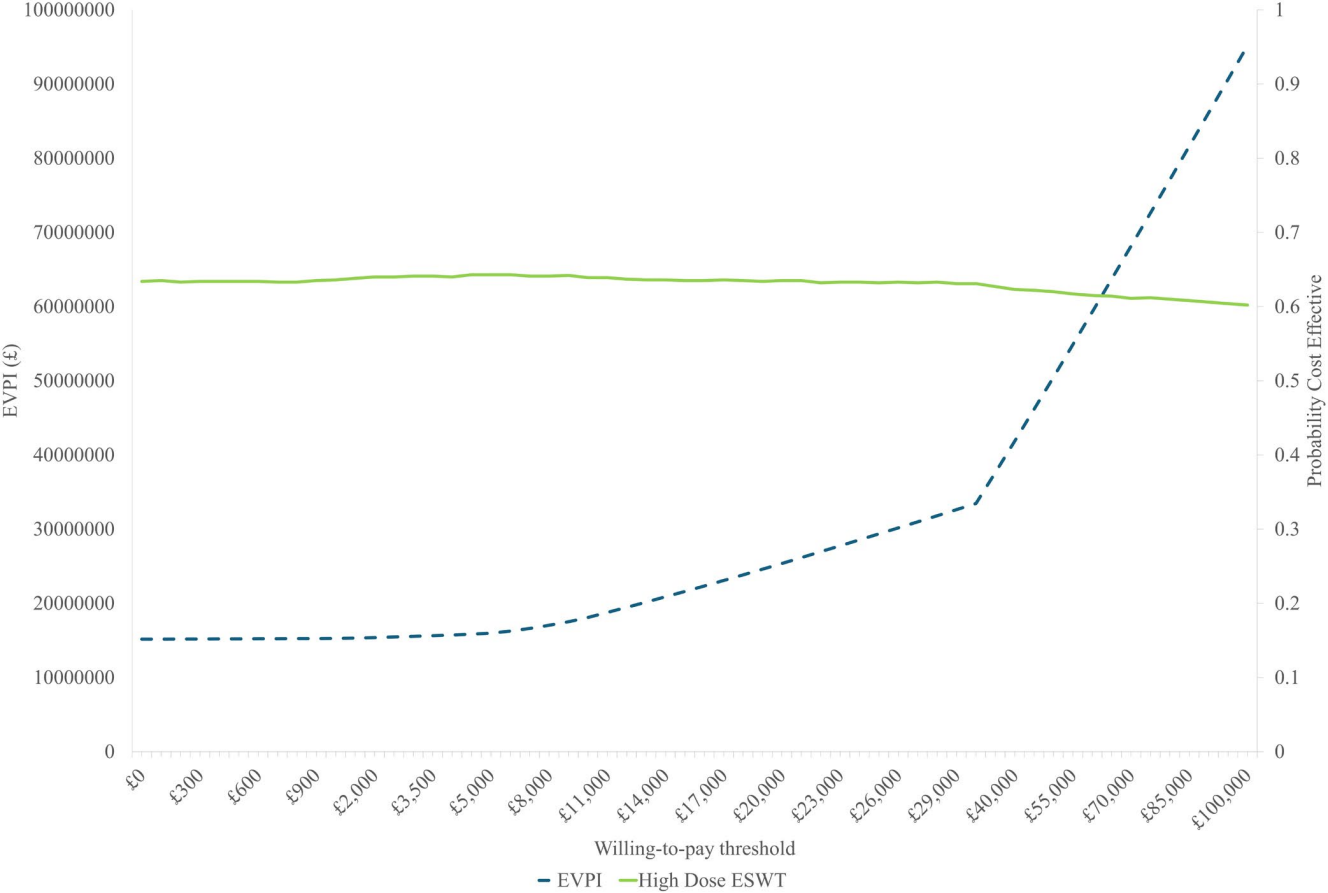
EVPI and cost‐effectiveness frontier. Blue dashed line = EVPI and willingness‐to‐pay threshold; Green solid line = probability treatment strategy (high dose ESWT) is cost‐effective and willingness‐to‐pay threshold.

At a threshold of £20 000 per QALY, the per‐patient EVPI was £24.56. The population EVPI, based on an incidence of 50 000 DFU per year, was £1 289 906.35. This rose to £2 579 812.69 for an annual incidence of 100 000 DFUs per year. Figure 4 shows that as the willingness‐to‐pay threshold increases, so does the EVPI.

The individual EVPPI, at a willingness‐to‐pay threshold of £20 000, was £7.87 for the effectiveness of a high dose ESWT, £0 for daily state‐specific costs and £3.56 for health state‐related utilities.

## 4 Discussion

Previous clinical trials and systematic reviews have consistently reported superior healing with ESWT, compared with standard care alone [19, 20, 68]. This is the first early economic evaluation of ESWT compared to standard care. Preliminary findings indicate the potential cost‐effectiveness of ESWT. High dose ESWT has the potential to be a dominant treatment strategy.

Our preliminary deterministic analysis suggested that high dose ESWT had the potential to be a dominant alternative (i.e., associated with lower costs and higher QALYs than low dose ESWT or standard care alone). Preliminary probabilistic sensitivity analysis suggested that high dose ESWT had the potential to be associated with a 60% chance of being cost‐effective.

Preliminary findings of an early value of perfect information analysis suggested that conducting a definitive study of ESWT compared to standard care could be good value for money (e.g., assuming NICE's £20 000 willingness‐to‐pay threshold for an additional QALY, preliminary population EVPI was between £1289906.35 to £2 579 812.69, depending on the effective target population size). Currently NICE does not require an EVPI analysis to be included in health technology assessments [69]. Despite this, EVPI is useful for guiding funding bodies and researchers on the acceptable cost of further research and the form of the research should take [52, 70]. In the context of DFU care, where current treatment costs the NHS in England an estimated £1billion per year, further investment into the evidence base for ESWT to reduce the burden of DFUs is possibly a worthwhile avenue of future research [4].

In our analysis, a Markov model was used to explore the decision problem. The model included four discrete health states and amalgamated people living with diabetes with an open wound on the foot for more than 4 weeks into one health state. This differs from other DFU models, in which DFUs are often split into ischaemic, infected, post‐debridement/minor amputation surgery, and so forth [27, 28, 29, 30, 31, 32, 33, 34, 35, 36, 37, 38, 39, 40, 41, 42, 43, 44, 45, 46, 47, 48, 49, 50]. Therefore, a potential weakness of the model could be that the use of one health state for an open DFU potentially oversimplified the decision problem and did not extend past 12 months. The influence of ulcer related characteristics known to affect healing [71, 72, 73, 74, 75, 76, 77, 78] were explored in the subgroup analysis. Increasing size and duration of the DFU reduced the NMB of ESWT, whereas infection did not seem to have an influence. Other economic evaluations have used a different health state for gangrene and ischaemia [28, 31, 46, 48, 50, 79]. Patients with ischaemia to the extent that is impacting healing require revascularisation, not ESWT, and therefore are not represented in this analysis. Further modelling, with sufficient data, should include the impact of ischaemia which does not require revascularisation to further explore the impact of this characteristic on cost outcomes. This would help refine the indication of ESWT.

Our preliminary findings should be interpreted with caution. The model's transition probabilities, utilities and costs are based on a small pilot trial of a relatively homogenous group of patients from a single centre in England. All patients were managed by a dedicated diabetic foot multidisciplinary team with regular contact with research clinicians. Outcomes in trials are often reported to be better compared to routinely collected data, for a multitude of reasons [80, 81]. This could mean that the clinical results extracted from the pilot RCT that informed this analysis may not directly translate into routine practice, and the analysis could have over‐estimated potential cost‐effectiveness estimates. Potential sources of bias from using the pilot trial results include selection, as it was a single centre study, performance, as the participants knew they were in a trial, and underpowering due to a small sample size. The effect of underpowering and extrapolation of data likely resulted in parameter uncertainty. This uncertainty was addressed through the probabilistic sensitivity analysis [23, 64]. A synthesis of pilot trial data and external data could have increased the external validity of the findings. However, a recent systematic review reported a significant heterogeneity between published ESWT trials and advised against combining data [19, 82]. Further multi‐centre studies are required to refine the model parameters and ensure the model is externally valid.

This early economic evaluation is the first to explore the potential cost‐effectiveness of ESWT in DFU healing. Preliminary findings suggest that high dose ESWT has the potential to be a dominant method of accelerating DFU healing. A definitive study must collect ulcer healing data, healthcare resource use, and utilities in a robust manner to test the external reliability of these findings.

## Funding

L.H. was funded by the NIHR Doctoral Research Fellowship NIHR301807. The views expressed are those of the author(s) and not necessarily those of the NIHR or the Department of Health and Social Care.

## Ethics Statement

The SOLEFUL study, which encompasses a pilot RCT, qualitative study and economic evaluation, was approved by the NHS Health Research Authority (IRAS: 311664, REC reference: 22/WA/0089) and sponsored by the Hull University Teaching Hospitals NHS Trust. The study was undertaken in line with the Declaration of Helsinki.

## Conflicts of Interest

The authors declare no conflicts of interest.

## Data Availability

The data that support the findings of this study are available from the corresponding author upon reasonable request.
